# Emerging evidence of Urolithin A in sports nutrition: bridging preclinical findings to athletic applications

**DOI:** 10.3389/fnut.2025.1585922

**Published:** 2025-05-16

**Authors:** Manda Wang, Liang Yu

**Affiliations:** ^1^School of Sport and Science, Beijing Sport University, Beijing, China; ^2^School of Strength and Conditioning Training, Beijing Sport University, Beijing, China

**Keywords:** Urolithin A, exercise, athletic performance, sports supplement, mitochondria

## Abstract

Urolithin A (UA), a gut microbiota-derived metabolite of ellagic acid and ellagitannins, is emerging as a novel sports nutrition supplement with multifaceted benefits. UA demonstrates osteoprotective effects through dual regulation of bone remodeling, delays joint degeneration by reducing synovial inflammation, and enhances muscle endurance and peak oxygen uptake. Mechanistically, UA enhances mitochondrial *β*-oxidation and potentially activating skeletal muscle adenosine 5′-monophosphate-activated protein kinase (AMPK) signaling and upregulating mitochondrial respiratory chain complex activity to exert ergogenic effects. Notably, the benefits of UA seem to be unaffected by diet. We propose once-daily dosing with tentative pre-exercise timing, though exercise’s impact on UA pharmacokinetics requires further investigation. While UA’s multifunctionality bridges gaps in conventional sports supplements, human trials validating ergogenic claims—particularly regarding muscle-specific adaptations and metabolic system modulation—remain critical. This synthesis positions UA as a promising yet incompletely characterized candidate in sports nutrition, warranting rigorous translational research.

## 1 Introduction

UA is a naturally occurring metabolite derived from dietary precursors, such as pomegranates, through the metabolic activity of the gut microbiota. UA plays a crucial role in improving mitochondrial function by stimulating mitophagy and inhibiting the age-related accumulation of dysfunctional mitochondria, thereby maintaining mitochondrial biogenesis and respiration within cells (1). Over the years, UA has been investigated as a therapeutic agent for a range of conditions, including neurodegenerative diseases (2, 3), osteoarthritis (4), and immune disorders (5). However, recent clinical studies have highlighted the capacity of UA to significantly improve muscle endurance and exercise performance in older adults (6). The molecular responses elicited by UA in human plasma and skeletal muscle resemble those induced by conventional exercise training (7), sparking considerable interest in the sports science community. In fact, as early as 2016, research demonstrated that UA supplementation enhanced running performance in both young and aged mice (1). Although the precise mechanism of UA as a sports supplement has yet to be fully elucidated, its ability to promote AMPK phosphorylation in skeletal muscle (1) and enhance adenosine triphosphate (ATP) production (8) may be key components of its efficacy.

Importantly, a series of standardized toxicological assessments confirmed the safety of UA, showing favorable profiles in both human and animal models, with minimal side effects (7, 9). The compound has also been positively reviewed by the U.S. Food and Drug Administration (9). Previous studies have primarily characterized the health benefits of UA in older adults. However, recent clinical trials indicate that UA supplementation may also contribute to improvements in muscle strength among young resistance-trained individuals (10), underscoring its potential applications in sports nutrition. Despite this promising evidence, UA has not yet been extensively studied as a sports supplement. A comparative analysis was conducted between UA and commonly used dietary supplements among athletes, focusing on five key dimensions: exercise performance-enhancing effects, potential physiological benefits, target populations, adverse effects, and usage restrictions and precautions (Supplementary Table S2). We found that UA is associated with fewer adverse effects, a broader spectrum of applicable populations, and the potential to integrate the benefits offered by multiple conventional supplements. These attributes collectively position UA as a highly promising candidate for future applications in the field of sports supplementation. However, no existing literature has systematically summarized the potential benefits of UA as a sports supplement on exercise performance, nor has it provided recommendations for supplementation strategies. Therefore, this review seeks to integrate existing clinical research on UA with its prospective applications in sports nutrition.

Through a comprehensive literature review utilizing databases such as PubMed, Google Scholar, Embase, and Web of Science, we synthesize current findings on UA’s absorption, metabolism, and health benefits in the context of its potential as a sports supplement, offering evidence-based recommendations for its application in athletic settings.

## 2 Production, absorption and metabolism of Urolithin A in humans

In 1980, UA was first identified as a metabolite of ellagic acid (EA) in rats (11). A seminal study subsequently demonstrated that the human gut microbiome converts ellagitannins (ETs), which are naturally occurring polyphenolic compounds, into UA, thereby bringing UA into the scientific spotlight (12). Moreover, two clinical studies revealed that the consumption of pomegranates (13) and nuts (14) significantly elevated UA levels in human plasma, leading to the conceptualization of UA as a potential dietary supplement.

### 2.1 Production

UA belongs to the urolithin (Uro) family, which is derived from ETs and EA through transformations mediated by the gut microbiota, producing UA in the colon.

A proper gut microbiome is a prerequisite for UA production. The concentration of UA in the human body fluctuates depending on factors such as age, health status, and dietary intake (15). The gut microbiota is pivotal in the metabolic conversion of ellagic acid into urolithins (16). However, the enzymes and associated genes governing the metabolic pathway from EA to UA remain incompletely understood. What is known so far is *Enterocloster* spp. and their induced urolithin C dehydroxylase operon are primary contributors to UA biosynthesis (17).

ETs and EA are found mainly in foods such as pomegranates, strawberries, and nuts. However, the conversion of these dietary precursors to UA is highly variable across individuals (18) and is observed in only approximately 40% of the population (19). Additionally, studies have shown that although cells treated with EA do not exhibit significant cytotoxicity, high concentrations of EA can induce cell proliferation (20). Therefore, it is recommended that colorectal cancer patients be cautious with foods high in EA (20). Notably, the same study also revealed that UA has anticancer properties (20). Accordingly, exogenous supplementation of UA not only maximizes its physiological benefits but also avoids the side effects associated with its digestion from food sources.

### 2.2 Absorption and metabolism

Once synthesized in the colon, UA can be absorbed through intestinal epithelial cells due to its lipophilic nature, subsequently entering the circulatory system via enterohepatic circulation. Upon hepatic absorption, UA undergoes phase II biotransformation, resulting in the formation of phase II metabolites. Within the range of 0.024 to 35 μM in the circulatory system, UA exists mainly as glucuronides and secondarily as sulfates (21). Recent studies indicate that after a single dose of UA, mice metabolize it rapidly within 3 h, producing three types of phase II metabolites: UA-3-O-glucuronide, UA-3-sulfate, and UA-sulfate glucuronide (20).

The primary biochemical process in Phase II metabolism is conjugation reaction, wherein endogenous or exogenous compounds are covalently linked to specific functional groups, resulting in the formation of conjugated metabolites. Generally, conjugation in Phase II metabolism reduces the bioavailability of phenolic compounds (22). For UA conjugated metabolites, glucuronidation enhances the molecule’s hydrophilicity, promoting renal excretion, whereas sulfation may attenuate the biological activity of the UA monomer. Several studies have noted that phase II metabolites of UA reduce its biological activity. The resistance of human colon cancer HT-29 cells to UA is attributed primarily to phase II metabolism (23, 24). Moreover, although UA inhibits tumor cell proliferation and exhibits anti-aging properties, UA-glucuronide and UA-sulfate do not influence tumor cell proliferation or aging cells (25).

**Figure 1:**
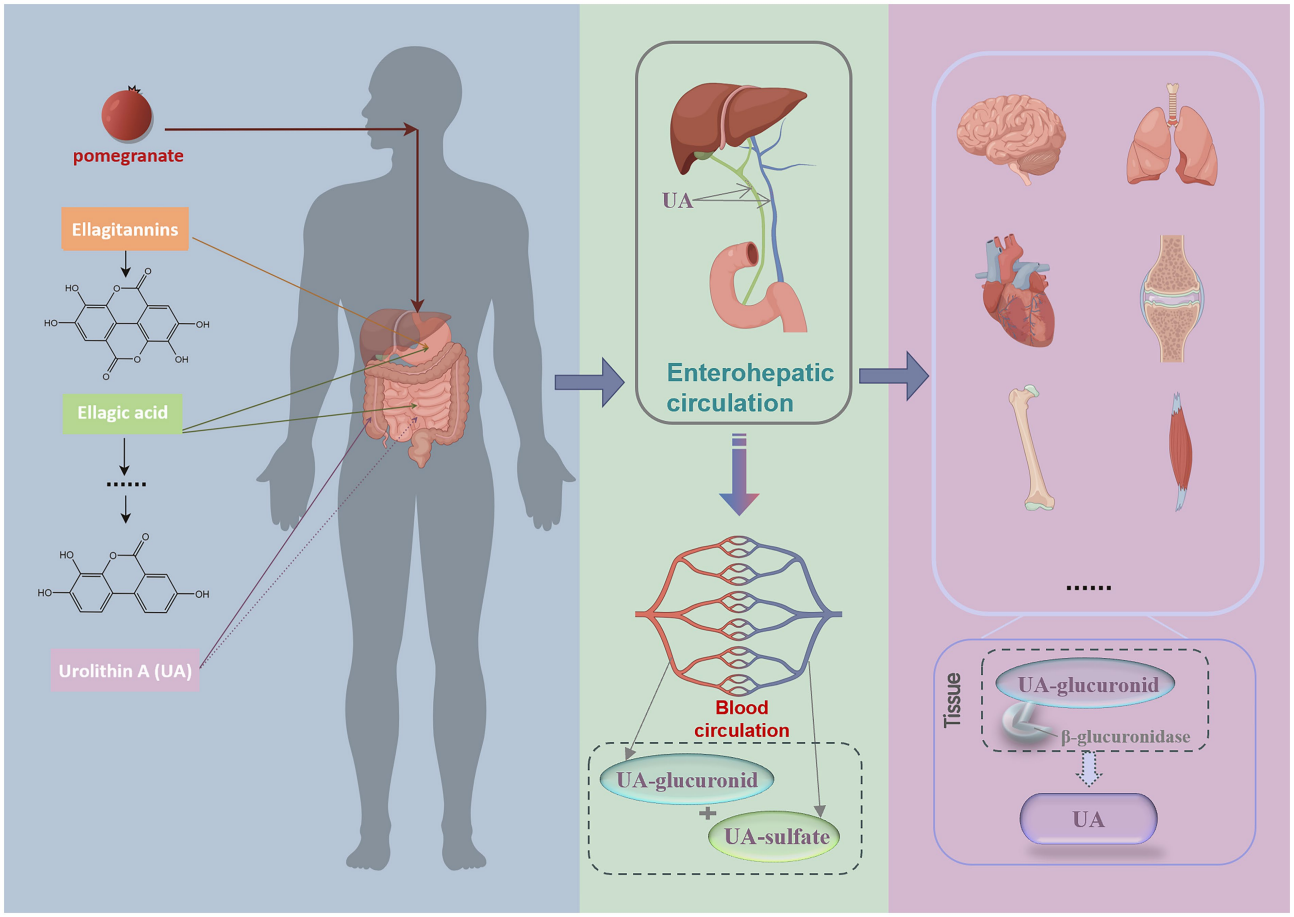
Process of UA exerting its biological activity after it is produced in the human body. Following pomegranate ingestion, gut metabolism produces ETs and EA, which are converted into UA in the colon through the gut microbiota. UA subsequently enters the bloodstream via enterohepatic circulation, primarily existing as its phase II metabolites, UA-glucuronides and UA-sulfates. Upon reaching tissues, it dissociates into UA monomers by enzymes and exerts its biological activity. This image was drawn by Figdraw.

However, how UA exerts its maximum biological activity in target organs and whether its form differs across various target organs remain unclear. Some scholars have proposed the “inflammatory dissociation” hypothesis (26, 27). This hypothesis posits that within inflamed tissues, a decoupling process in the inflammatory microenvironment reconverts UA-glucuronide conjugates into UA monomers. This hypothesis was also validated in a lipopolysaccharide (LPS)-induced systemic inflammation rat model (28). But the triggering conditions under which phase II metabolites are converted back into UA monomers have not yet been reported, indicating a need for more research in this area. Furthermore, studies have shown that following the consumption of ellagitannin-rich pomegranate extract, high concentrations of UA are detected in the prostate, gut, and colon of prostate cancer model mice, whereas elevated levels of UA-sulfate and UA-glucuronide are detected in the liver and kidneys (29). Additionally, plasma samples from mice collected 1 and 6 h after collection presented a decrease in UA monomers from 77.2 to 65.7%, accompanied by an increase in phase II metabolites (30), indicating that UA progressively forms conjugated metabolites after entering the plasma. In elderly individuals administered a single 2000 mg UA capsule, the majority of UA detected in skeletal muscle was in its monomeric form, with only a few subjects showing trace amounts of UA-glucuronide (7). We therefore hypothesize that following entry into the bloodstream, UA is converted into less biologically active phase II metabolites, which subsequently dissociate into more active UA monomers within tissues and inflammatory microenvironment (Figure 1).

### 2.3 Safety of human ingestion

Considering the strict microbiome conditions necessary for UA production, the most efficient approach to harness the biological effects of UA is through *in vitro* synthesis followed by supplementation. Thus, rigorous safety assessments of UA supplementation are essential.

In 2019, Andreux et al. (7) reported the first human clinical trial of UA supplementation. In this trial, researchers administered acute single doses (250, 500, 1,000, 2000 mg/day) and chronic doses of oral UA for 4 weeks (250, 500, 1,000 mg/day, 7 days/week) to healthy, sedentary elderly individuals, assessing both safety and bioavailability. The participants underwent comprehensive physical examinations before and after the intervention, including assessments of major body systems/areas: skin and mucous membranes, ears, nose, and throat, lungs, heart, gastrointestinal, and nervous systems. In addition to normal physical examination findings, electrocardiogram indicators and liver and kidney function-related biochemical markers, as well as blood and urine test results, were within normal ranges, confirming the absence of toxicity across all tested UA doses. The study also revealed that UA bioavailability remained unaffected by the co-consumption of high-protein yogurt. Similarly, Ryu et al. (1) reported that UA does not extend the nematode lifespan through alterations in bacterial counts, metabolism, or food intake. These findings suggest that UA supplements exert their effects independently of dietary factors. However, to date, no such adverse effects have been reported in human clinical trials involving UA supplementation, further supporting its favorable safety profile.

Following a 4-month long-term supplementation study in elderly individuals, Singh et al. (6) found that both 500 mg/day and 1,000 mg/day UA were well tolerated and exhibited high safety profiles. Some participants reported minor adverse effects, primarily involving skeletal muscle and connective tissue, which researchers attributed to muscle biopsies rather than the UA itself. In conclusion, oral UA supplementation, whether administered acutely or chronically at both low and high doses, demonstrate considerable safety. Given that dietary factors do not significantly influence UA supplementation, the associated safety concerns are minimized. Nonetheless, research on potential interactions between UA and other drugs remains limited, warranting further investigation prior to broader application and promotion.

## 3 Effects of UA on athletic performance

### 3.1 Optimizing the force transmission of the kinematic chain

Force transmission within the human movement chain is foundational to the execution of movement. Optimizing and activating each component within this chain leads to improved force transmission efficiency, enhancing athletic performance and reducing injury risk. Recent clinical trials have demonstrated that UA positively impacts the fundamental elements of the kinematic chain.

#### 3.1.1 Improving bone health

Not all exercise modalities have been demonstrated to exert beneficial effects on skeletal health. Athletes participating in endurance disciplines—such as long-distance running, road cycling, and swimming—frequently present with reduced bone mineral density relative to athletes from other sports, control groups, or established population norms. Moreover, bone mass may not fully recover following retirement from competitive athletics (31, 32). These observations highlight the need to prioritize skeletal health in athletic populations. Considering the bone tissue is modulated by nutrition, dietary supplementation may represent a pivotal strategy for supporting long-term skeletal health in this demographic.

UA enhances bone health through two primary mechanisms: promoting osteogenesis and inhibiting osteoclast-mediated bone resorption. UA has been shown to increase calcium salt deposition in mouse bone marrow mesenchymal stem cells and promote osteoblast differentiation in osteopenic mice (33), indicating its potential for enhancing osteogenesis. With aging, bone resorption gradually surpasses bone formation, resulting in bone loss. Osteoclasts are recognized as the exclusive cells responsible for bone resorption (34). UA inhibits osteoclastogenesis induced by receptor activator of nuclear factor kappa-B ligand in a concentration-dependent manner, effectively mitigating bone loss (35). In conclusion, UA promotes new bone formation by enhancing calcium deposition and reducing the resorption of existing bone simultaneously. These findings suggest that UA supplementation enhances bone density, hardness, and stiffness, resulting in stronger, more resilient bones with denser trabecular architecture, thereby better balancing internal bone forces.

Long-term skeletal health in athletes hinges on two primary considerations: first, optimizing bone mass accrual during the developmental years; and second, addressing the difficulty of eliciting adequate and sustained osteogenic stimuli after peak bone mass has been attained, in order to mitigate age-related bone loss (32). UA, owing to its dual role in promoting osteogenesis and inhibiting bone resorption, presents a promising candidate for the sustained maintenance of skeletal health in athletes. Moreover, its therapeutic potential may extend to injury-related conditions, such as stress fractures, by facilitating skeletal repair and recovery. However, the physiological effects of UA on bone health in athletic populations have yet to be substantiated by clinical trials, underscoring a critical gap in the current literature that merits further investigation.

#### 3.1.2 Enhancement of the joint chain

The rigid structures in the joints, such as bones and cartilage, can transmit force. The state of the joint chain affects the mechanical distribution during exercise, which in turn influences the state of movement and athletic performance.

A recent human trial has confirmed the potential of UA in enhancing joint mobility and mitigating joint structural damage (4). Researchers isolated primary human chondrocytes (HCs) from the knees of healthy individuals and treated them with UA at two concentrations (6.25 μM and 12 μM) for 24 h. Both doses led to an increase in maximal respiration and ATP-linked respiration in HC cells, while mitochondrial biogenesis and oxidative phosphorylation gene expression remained unchanged, suggesting that UA specifically enhances mitochondrial respiration to improve chondrocyte metabolic function. Furthermore, the study confirmed that UA enhances mitophagy through activation of the PTEN-induced putative kinase 1 (PINK1)- Parkinson protein (Parkin) pathway and modulates phosphorylated ubiquitin levels. These findings indicate that UA supplementation may provide additional benefits for joint health in the general population.

**Figure 2:**
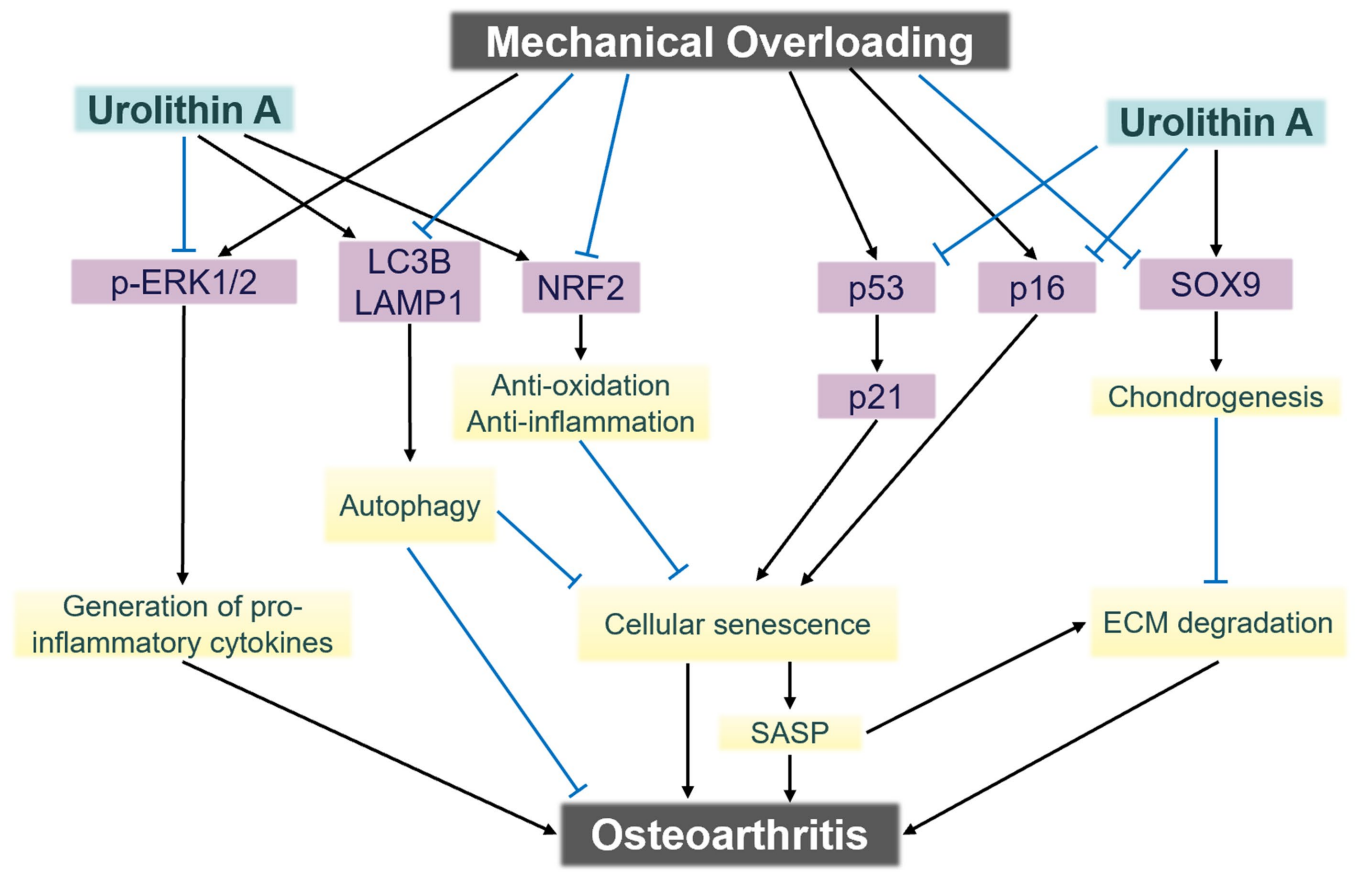
The signaling mechanisms underlying chondrocyte damage induced by mechanical overloading. Urolithin A exerts chondroprotective effects against mechanical trauma via multi-pathway modulation. ECM, Extracellular matrix; SASP, senescence-associated secretory phenotype. Adapted from He et al. (36), licensed under CC BY 4.0.

Dynamic compressive loading during physical activity represents a key biomechanical factor implicated in cartilage degeneration. He et al. (36) modeled mechanical loading conditions analogous to those encountered during walking and applied controlled mechanical injury to healthy human cartilage tissue, subsequently administering UA treatment. When the mechanical load reached a level that caused cartilage cell damage, markers of mitochondrial autophagy decreased, accompanied by a decline in chondrogenic gene expression (36). Treatment with 10 μM UA did not alter chondrogenic gene expression but led to a reduction in pro-inflammatory cytokines and cartilage-degrading enzymes, alongside an upregulation of chondrogenic markers Collagen Type II and Aggrecan (36). These findings suggest that UA can mitigate cartilage damage induced by mechanical stress. Furthermore, the study suggested that this protective effect is mediated via the upregulation of mitochondrial autophagy (36). Notably, the results demonstrated that UA exerts its effects by downregulating microtubule-associated proteins 1A/1B light chain 3B and Lysosomal-associated membrane protein 1 expression, without significantly altering PINK1 and Parkin protein levels (36). The researchers further investigated additional molecular mechanisms underlying UA’s effects (Figure. 2), including the involvement of p-extracellular regulated protein kinases signaling (36). Although preliminary, these findings open multiple promising avenues for future research in this domain.

#### 3.1.3 Strengthening muscle strength and endurance

With respect to force transmission in the kinetic chain, the direct impact of UA lies in its ability to increase muscle strength. Singh et al. (6) evaluated leg muscle strength of healthy elderly individuals by isokinetic Biodex dynamometer strength testing after 4 months of UA supplementation at 500 mg/d and 1,000 mg/d. The results demonstrated that both dosage levels significantly enhanced maximum torque during knee flexion, as well as the average peak torque of the hamstring muscles in older adults. The increase in maximum torque reflects an improvement in the hamstrings’ peak force output during a single contraction, indicative of enhanced maximal muscle strength. In parallel, the elevation in average peak torque suggests an enhanced capacity to maintain high force output across repeated contractions, reflecting improvements in muscular endurance and resistance to neuromuscular fatigue.

However, the trial conducted among young resistance-trained individuals yielded limited improvements in performance outcomes. Zhao et al. (10) reported that 8 weeks of UA supplementation (1,000 mg/day) did not produce statistically significant improvements in one-repetition maximum (1RM) performance for the bench press or squat in young male resistance-trained athletes, although a non-significant upward trend was noted. In contrast, a statistically significant increase in maximal voluntary isometric contraction (MVIC) of the quadriceps was observed both within and between groups. MVIC represents the peak force generated during static isometric exertion, and its enhancement is primarily attributed to neural adaptations (37). These neural adaptations tend to develop more rapidly in response to isometric training but exhibit limited transferability to dynamic movements such as the bench press or squat, which involve complex multi-joint coordination and precise temporal control (38). The observed increase in MVIC in the absence of changes in 1RM suggests that neural adaptations may have occurred in response to UA supplementation, whereas structural adaptations essential for dynamic performance—such as muscle hypertrophy, tendon stiffness, and neuromuscular coordination—may have lagged, potentially due to insufficient movement-specific training stimuli. Considering that the participants were already resistance-trained, additional structural adaptations may necessitate a more substantial training stimulus. The potential for further 1RM enhancement may be constrained without extended UA supplementation, increased training intensity, and exercise protocols tailored to specific movement patterns. UA supplementation may yield more pronounced effects in novice individuals, who possess greater capacity for both neural and structural adaptations.

Recently, a human trial has characterized the enhancement of muscular endurance through UA supplementation. This research involving 20 young male resistance-trained individuals, 8 weeks of UA capsule supplementation, in combination with resistance training, significantly improved performance in Repetitions to Failure (10). In a study by Singh et al. (6), although there were no statistically significant differences between the UA group (1,000 mg/day) and the control group in peak oxygen uptake and performance on the 6-min walk test (6MWT), the UA group showed significant improvements compared to their own baseline.

UA has demonstrated remarkable potential in enhancing a key gold-standard indicator of aerobic endurance — maximal oxygen uptake (VO₂_max_). Both human and animal studies have demonstrated that UA supplementation increases cardiac ejection fraction (39, 40), suggesting a potential for enhancing stroke volume and overall cardiac output. Additionally, UA promotes skeletal muscle angiogenesis through modulation of the silent information regulator 1 and peroxisome proliferator-activated receptor-gamma coactivator-1-alpha signaling pathways, resulting in increased capillary density and improved muscular endurance (8). A separate human study also found that ingesting 1,000 mg of pomegranate extract, which is rich in UA, prior to a single bout of high-intensity exercise, significantly improved blood flow and vascular diameter in resistance-trained individuals (41). Collectively, these results suggest that UA not only enhances oxygen delivery through improved cardiovascular function but also augments oxygen utilization at the muscular level — both of which are critical determinants of increased VO₂_max_.

Interestingly, the increasement in endurance appears to be non-specific to particular muscle groups. Research conducted by Liu et al. (42) found that UA supplementation significantly improved the endurance of both the dorsal interosseous muscles of the hand and the tibialis anterior muscles of the leg, which are two muscle groups that differ considerably in function and anatomical structure. The tibialis anterior contains approximately 75% type I fibers, favoring slow-twitch fibers, and is involved in exercise testing, whereas the dorsal interosseous muscles consist of approximately 50% type II fibers, with no direct involvement in movement. These findings suggest that UA may improve overall muscular endurance irrespective of whether the muscle group is classified as an advantageous one in exercise.

### 3.2 Cardiovascular protection

#### 3.2.1 UA improves cardiac pump function

The left ventricle must generate adequate contractile force to propel blood into the systemic circulation. This requirement becomes particularly critical during high-intensity exercise when muscle demand for blood volume increases proportionally. Consequently, the left ventricle must deliver an increased volume of blood to active muscle tissue, placing substantial demands on the pumping capacity of the heart.

In the early stages of endurance exercise, physiological responses—including sympathetic nervous system activation and catecholamine release—elevate cardiac output to meet the demands of exercising organs (43). However, extended endurance exercise may result in reduced cardiac function, a phenomenon known as “cardiac fatigue” (43).

After endurance training, a decrease in the left ventricular ejection fraction (EF) is commonly observed in both untrained individuals and experienced athletes (43). An 8-week UA intervention significantly improved the early to late diastolic transmitral flow velocity ratio, fractional shortening, and EF in aging rats (44). These findings suggest that UA supplementation has significant potential for enhancing cardiac pumping function. Thus, postexercise UA supplementation may alleviate the “cardiac fatigue” associated with prolonged physical activity.

Nonetheless, some studies have reported negative results. Four-week UA supplementation (500 mg/d) did not significantly improve the EF in patients with heart failure or reduce the ejection fraction (40). This lack of improvement could be attributed to the low dosage of UA and the brief duration of the intervention. In most human trials concerning UA, the doses that exhibit significant physiological effects are typically approximately 1,000 mg/d.

#### 3.2.2 Reducing the incidence of exercise-induced cardiac injury with UA

Regular long-term exercise induces structural, functional, and electrical adaptations in the heart; however, not all of these adaptations are beneficial. For example, arrhythmias may develop as a result of increased vagal tone and left atrial enlargement. Besides structural adaptations, exercise significantly elevates circulating biomarkers associated with cardiovascular disease, such as creatine kinase, cardiac troponin, and B-type natriuretic peptide (BNP) (45). While the sources of these circulating molecules remain unclear, their elevation may be linked to skeletal muscle injury and myocardial stress activation, signaling myocardial damage and cardiac fatigue. Current studies indicate that UA intervention significantly reduces elevated creatinine kinase-MB, cardiac troponin and BNP levels in rat cardiomyocytes under metabolic stress (46, 47). Additionally, UA treatment mitigated the expression of proinflammatory factor fractalkine in rat myocardium, prevented early inflammatory responses in cardiomyocytes during hyperglycemia, and restored calcium dynamics and contractility (48). Thus, UA supplementation during prolonged exercise is likely to attenuate cardiac stress responses and diminish the degree of cardiac fatigue.

Myocardial fibrosis, characterized by the accumulation of scar tissue in the myocardium or valves, has also been observed in some lifelong endurance athletes (45). The exact mechanism remains incompletely understood. Chen et al. (49) have confirmed that *in vitro* treatment with UA can reduce the expression levels of fibrosis markers including vimentin, discoidin domain receptor tyrosine kinase 2, tensin and Alpha-smooth muscle actin in myocardial tissues, and inhibit the transition of cardiac fibroblasts to a fibrotic state through nuclear factor erythroid 2-related factor 2 signaling pathway. These findings suggest that appropriate UA supplementation may delay or prevent myocardial fibrosis in lifelong athletes, but further experimental evidence is needed to support this finding. Clinical investigations into the cardiovascular benefits of UA remain limited, with the majority of current evidence originating from *in vitro* and animal models. The present findings merely indicate the potential of UA supplementation to improve cardiac function in the context of structured exercise.

### 3.3 Accelerating the recovery of exercise-induced fatigue

#### 3.3.1 Role of UA in alleviating inflammatory responses

The overtraining-cytokine hypothesis proposes that prolonged stress, insufficient recovery periods, and disrupted sleep patterns may trigger sustained cytokine release, leading to chronic systemic inflammation, which is hypothesized to underlie the development of chronic fatigue in athletes (50, 51). In recent years, polyphenolic compounds derived from pomegranate have been investigated as dietary supplements aimed at alleviating exercise-induced fatigue (52), with studies highlighting the anti-inflammatory properties of pomegranate extracts.

High-intensity or prolonged physical activity may induce mechanical damage to muscle fibers, subsequently eliciting localized inflammatory responses (53). Damaged muscle cells initiate immune responses and stimulate the release of pro-inflammatory cytokines (54). C-reactive protein (CRP), a widely recognized biomarker of systemic inflammation, has been reported to decrease significantly following UA supplementation (6, 10). Zhao et al. (10) observed that CRP levels significantly increased in both the control and UA-supplemented groups compared to baseline among resistance-trained individuals, likely due to incomplete recovery during the 8-week training intervention, which may have led to persistent low-grade inflammation. However, compared to the control group, participants receiving UA exhibited significantly lower plasma CRP concentrations (10), suggesting that UA supplementation may help attenuate exercise-induced inflammatory responses.

Typically, moderate inflammation is viewed as a component of athletic adaptation, while suppressing inflammation might interfere with the adaptive response to training. However, in specific scenarios, such as those involving participation in multiple events within short intervals, athletes might prioritize rapid recovery or reduced soreness over long-term adaptation. The accumulation of inflammatory compounds can stimulate fluid retention and activate nociceptors—both of which are closely associated with the sensation of pain (55), rendering the use of anti-inflammatory agents necessary. The pain-relieving effect of UA has been characterized by animal experiments. D’Amico et al. (4) reported that an 8-week UA intervention led to a significant reduction in pain responses in a murine model of osteoarthritis. Furthermore, they validated that this analgesic effect was linked to a reduction in inflammation, a mechanism corroborated by multiple studies demonstrating UA’s anti-inflammatory properties in chondrocytes (56, 57). However, it is crucial to acknowledge that current evidence is primarily derived from animal studies, and the analgesic effects of UA in humans remain to be clinically substantiated.

#### 3.3.2 UA alleviates oxidative stress

The oxidative stress response generated during exercise is linked to recovery from muscle fatigue. The production of reactive oxygen species (ROS) induced by moderate-intensity exercise plays a crucial role in skeletal muscle adaptation to exercise. Conversely, excessive levels of oxidants in skeletal muscle, often linked to prolonged exercise, can impair proteins critical to excitation–contraction coupling, thereby inducing muscle fatigue and diminishing the physiological advantages of ROS production during moderate-intensity exercise (58). UA, recognized as an antioxidant, can neutralize free radicals and reduce their harmful effects on cells. Research has demonstrated that UA intervention decreases ROS production by inhibiting the activity of NADPH oxidase, a key source of cellular ROS, and subsequently reduces the production of proinflammatory mediators through the regulation of the phosphatidylinositol 3-kinase / protein kinase B / nuclear factor kappa B and c-Jun N-terminal kinase / activator protein 1 signaling pathways (59). Additionally, UA can increase the synthesis of several antioxidant molecules in animals, including glutathione, catalase, and glutathione peroxidase (60). This ability helps reduce oxidative stress within muscle cells, thereby preserving their structural integrity and function and ultimately contributing to overall muscle health.

### 3.4 Enhancing mitochondrial health to improve energy metabolism

A key physiological effect of UA lies in its ability to modulate mitochondrial function. Extensive preclinical and clinical studies, including trials across diverse species, have consistently reported that UA enhances mitochondrial health primarily through mitophagy (1, 6, 7, 42). Mitophagy serves to maintain mitochondrial quality within cells, thereby augmenting mitochondrial respiratory capacity. Multiple studies have indicated that, following mitophagy induction, UA promotes mitochondrial biogenesis, accelerates mitochondrial turnover and restores mitochondrial function (5, 61).

As the primary energy currency for various tissues, ATP, which is produced by mitochondria, is essential for muscle contraction and athletic performance. Research has shown that UA can facilitate the formation of mitochondrial respiratory chain complexes and enhance their function, increasing ATP synthesis in muscle cells (6). The antioxidant properties of UA can also reduce free radical production, protect mitochondria from oxidative stress-induced damage, and enhance the efficiency of energy production. Collectively, these mechanisms enable UA to augment energy availability in muscle cells, thereby enhancing both endurance and strength performance.

Lipids serve as an important energy source during exercise, and enhanced fatty acid oxidation capacity contributes to improved exercise performance (62). Acylcarnitine is a form that enables fatty acids to enter mitochondria for oxidation, the plasma acylcarnitine levels in human circulating blood significantly decrease after UA supplementation (6, 7), indicating an enhancement in mitochondrial *β*-oxidation capacity. Research by Andreux et al. (7) also demonstrated that UA has a particularly significant effect on short-chain acylcarnitine, an intermediate in the fatty acid oxidation process, indicating increased efficiency in fatty acid oxidation (63).

In summary, UA enhances energy availability in muscle cells by promoting mitochondrial turnover, boosting mitochondrial activity, facilitating ATP synthesis, and increasing fatty acid oxidation rates.

## 4 Recommendations for supplementing the UA during exercise

The pharmacokinetics of UA in humans have been well characterized. In the single ascending dose study, UA demonstrated bioavailability in plasma across all the tested doses ranging from 250 to 2,000 mg (7). With continuous supplementation over 28 days, the maximum plasma concentration and total exposure to UA increase in a dose-dependent manner (7). Moreover, the health benefits of UA also exhibit dose dependency. For instance, in elderly individuals, mitochondrial markers in muscle did not significantly change with a 250 mg/day intervention, but notable improvements were observed at 500 mg/day and 1,000 mg/day (7). Therefore, achieving the desired benefits of UA supplementation in the context of exercise necessitates an appropriate dosage.

The half-life of UA and UA-glucuronide ranges from 17 ~ 22 h, whereas the half-life of UA-sulfate is slightly longer, varying between 25 ~ 58 h (7). Thus, a single daily dose of UA is sufficient. Current studies often use 500 mg/day and 1,000 mg/day as intervention doses. While both doses are generally safe, 1,000 mg/day tends to provide greater health benefits (6, 7). Specifically, with respect to exercise performance, a dosage of 1,000 mg/day significantly enhances muscle endurance and VO_2peak_ compared with baseline in elderly individuals, whereas 500 mg/day does not yield comparable benefits (6). Therefore, we recommend a dosage of 1,000 mg/day for UA as an exercise supplement.

UA and its phase II metabolites (UA-glucuronide and UA-sulfate) exhibit similar pharmacokinetic profiles in the human body. Following a single dose, plasma concentrations peak at approximately 6 h, with phase II metabolites predominantly present. At 8 h post-administration, UA is detected in skeletal muscle primarily in its monomeric form (7). This suggests a delay between UA entry into the circulation and its arrival at target tissues, where it dissociates into the monomer to exert its effects. Therefore, supplementation with UA 6 to 8 h before exercise may optimize its benefits. It is worth noting that, to date, no studies have investigated the impact of exercise on the absorption and metabolism of UA. Whether exercise influences the dissociation of phase II metabolites remains to be further explored.

Existing human studies on UA interventions have focused predominantly on elderly populations. Research on children, adolescents, and young adults is lacking. Different metabolic states may lead to variations in UA absorption and metabolism (Supplementary Table S1), and optimal doses may differ. Further investigations are needed to address these knowledge gaps.

## 5 Conclusion

UA is a beneficial natural metabolite endogenously produced in the human body. However, owing to variations in the composition of the gut microbiota, only a limited percentage of individuals can effectively synthesize UA, with UA production fluctuating with age and environmental influences. Therefore, to use UA as a dietary supplement, relying solely on food conversion is insufficient, making externally synthesized UA supplements necessary. The safety of UA supplements has been well established, even at higher dosages, with no observed side effects. UA supplementation is deemed suitable for both younger and elderly populations. As a sports supplement, UA enhances the quality of bones, joints, muscles, and the kinetic chain. It also possesses mild anti-inflammatory and antioxidant properties, helping to alleviate muscle fatigue and potentially offering cardioprotective effects during prolonged physical exertion. Notably, UA supplementation is unaffected by high-protein diets or food intake levels, avoiding potential dietary interactions. However, the impact of exercise on UA absorption and metabolism remains unexplored and studies on its application in athletes are still scarce. Further research is warranted to establish comprehensive guidelines on UA supplementation tailored to specific health and performance goals.
