# Supplementary material for: Emerging evidence of Urolithin A in sports nutrition: bridging preclinical findings to athletic applications
Source: Front Nutr. 2025 May 16;12:1585922. doi: 10.3389/fnut.2025.1585922 (PMC12122305; doi:10.3389/fnut.2025.1585922)
Supplement: Supplementary file 1 [file Table_1.pdf]

frontiers

Supplementary Material

1 Supplementary Table

Table 1 UA intervention methods and corresponding physiological effects in human experiments

Study	Subject characteristics and average age (year)	Gender and number of subjects	Duration of intervention	UA intake dosage and method	Physiological effects
**Andreux 2019(1)**	Healthy, sedentary, elderly<br>68±4.6	male (n=3) + female (n=3)	Single dose	250 mg/2000 mg<br>Oral soft gels	Untested
	Healthy, sedentary, elderly<br>a. 68.3±5.0<br>b. 67.5±4.7	male (n=3) + female (n=3)	Single dose	a. 500 mg<br>b. 1000 mg<br>Oral soft gels/Mix into yogurt foods	Untested
	Healthy, sedentary, elderly<br>67.2±5.9	male (n=3) + female (n=6)	28 days	250 mg/d<br>QD<br>Oral soft gels	Mitochondrial Health (-/-)
	Healthy, sedentary, elderly<br>65.4±3.7	male (n=3) + female (n=6)	28 days	500 mg/d<br>QD<br>Oral soft gels	Mitochondrial biosynthesis in skeletal muscle ↑<br>Fatty acid oxidation ↑
	Healthy, sedentary, elderly<br>64.8±4.2	male (n=3) + female (n=6)	28 days	1000 mg/d<br>QD<br>Oral soft gels	Mitochondrial biosynthesis in skeletal muscle ↑<br>Skeletal muscle mitochondrial autophagy↑<br>Fatty acid oxidation ↑
**Liu 2022(2)**	Healthy, elderly<br>64.8±4.2	male (n=6) + female (n=27)	4 months	1000 mg/d<br>QD<br>Oral soft gels	Skeletal muscle endurance ↑<br>Plasma inflammatory level ↓<br>Mitochondrial metabolism ↑<br>Maximum ATP production (-/-)
**Singh 2022(3)**	Untrained, overweight, middle-aged<br>51.0±7.2	male (n=11) + female (n=18)	4 months	500 mg/d<br>QD<br>Oral soft gels	Leg muscle strength ↑<br>VO_{2peak} (-/-)<br>VO_{2max} (predicted) (-/-)<br>Physical performance (-/-)<br>Fatty acid oxidation ↑<br>Plasma inflammatory marker content (-/-)
	Untrained, overweight,	male (n=12) + female (n=18)	4 months	1000 mg/d	Leg muscle strength ↑

	middle-aged<br>52.1±5.6			QD<br>Oral soft gels	VO_{2peak} (-/-)*<br>VO_{2max} (predicted) (-/-)*<br>Physical performance (-/-)*<br>Fatty acid oxidation (-/-)<br>Plasma inflammatory marker content ↓
**Nishimoto 2023(4)**	UA non/low producers with poor endothelial function<br>53.1±5.8	male (n=9) + female (n=3)	12 weeks	10 mg/d<br>QD<br>Oral soft gels	Vascular endothelial function (-/-)<br>Overall gut microbiome characteristics (-/-)<br>Diversity of gut microbiota alpha (-/-)<br>4 gut microbial genera changed
	UA non/low producers with poor endothelial function<br>54.1±5.6	male (n=7) + female (n=4)	12 weeks	50 mg/d<br>QD<br>Oral soft gels	Vascular endothelial function (-/-)<br>Overall gut microbiome characteristics (-/-)<br>Diversity of gut microbiota alpha ↑<br>9 gut microbial genera changed
**Jamialahmadi 2024(5)**	Patients with heart failure and reduced ejection fraction<br>38.3±5.4	male (n=1) + female (n=9)	4 weeks×2, interval 2 weeks washout period	500 mg/d<br>250 mg BID<br>Oral soft gels	Various indicators of cardiac function (-/-)<br>HDL-C↑
**Denk 2024(6) (Conference Summary)**	Healthy, middle-aged and elderly<br>45-70	25 in total, gender ratio unknown	28 days	1000 mg/d<br>QD<br>Oral, unknown form	Immune remodeling<br>Mitochondrial quality ↑<br>Inflammation level ↓
**Zhao 2024(7)**	Healthy, long-term resistance trainer<br>24.1 ± 1.59	male (n=20)	8 weeks	1000 mg/d<br>500 mg BID<br>Oral soft gels	1RM bench press and squat frequency (-/-)<br>Maximum Voluntary Isometric Contraction (MVIC) and repetitions to failure (RTF) performance ↑<br>3-MH ↓ / CRP ↓ / IL-6 (-/-) / SOD ↓

The subjects represented in the table include only those who underwent UA interventions, excluding the control groups from each study. The symbol "↑" indicates an increase, while "↓" signifies a decrease; "(-/-)" denotes no statistically significant change; "*" indicates statistically significant difference from baseline. All studies, except for Nishimoto 2023(4), Jamialahmadi 2024(5) and Zhao 2024(7), underwent and passed drug safety evaluations.

Table 2 Functional comparison between UA and dietary supplements commonly used by athletes

Supplement	Exercise performance enhancing benefits	Potential benefits	Target population	Adverse effects	Usage restrictions & precautions
**Urolithin A**	①Enhance muscle strength (neural fitness) and endurance.<br>②Reduce the level of oxidative stress during training.<br>③Improve mitochondrial quality and regulate energy metabolism.	①Promoting osteogenesis and inhibiting osteoclast-mediated bone resorption.<br>②Enhancing joint mobility, mitigating joint structural damage and relieving joint pain.<br>③Improve cardiac pumping function and may reduce cardiac fatigue.	Almost unlimited	Some participants reported minor adverse effects, primarily involving skeletal muscle and connective tissue, which researchers attributed to muscle biopsies rather than the UA itself.	When it comes to increasing maximal muscular strength, UA supplementation appears to be more effective for exercise novices and older adults, while experienced trainees may require additional sport-specific training to achieve significant gains.
**Creatine Monohydrate(8-11)**	①Increase strength and power for high-intensity, short-duration activities (e.g., weightlifting, sprinting).<br>②May help delay fatigue and improve training quality.	①Potentially support muscle recovery.<br>②Some research also points to neuroprotective and cognitive benefits.	①Strength athletes and sports participants.<br>②Individuals engaged in short, high-intensity efforts.	Weight gained in a short time primarily due to water retention.	①Those with kidney dysfunction or related conditions should use it cautiously.<br>②It is important to stick to the recommended dosage to avoid overdose.
**β-Hydroxy β-Methylbutyrate (HMB)(12-14)**	①Helps reduce muscle protein breakdown during exercise and promotes muscle recovery.<br>②May assist in enhancing strength and endurance through	①Aids in maintaining and increasing lean body mass.<br>②In older adults, it may help improve muscle quality and reduce the risk of falls associated with muscle loss.	①Individuals performing resistance training.<br>②Older adults or those in recovery phases.	Generally safe.	Long-term high doses should be avoided due to potential adverse effects.

	improved training adaptations.				
**Vitamin D(15, 16)**	Supports muscle contraction and overall strength (This view is controversial).	①Enhances bone health and may prevent osteoporosis.<br>②Modulates immune function, potentially reducing the risk of infections.<br>③Some studies have shown a reduction in falls and related injuries.	①Individuals with a vitamin D deficiency.<br>②Older adults. Athletes and those regularly train outdoors.	Excessive or prolonged intake may lead to hypercalcemia, accompanied by symptoms like nausea, vomiting, and kidney calcification.	High doses require medical supervision to avoid long-term overconsumption.
**Probiotics(17-19)**	No direct benefits for improving performance.	①May indirectly support post-exercise recovery by improving gut microbiota balance and enhancing immune response.<br>②Certain strains help reduce gastrointestinal discomfort associated with strenuous exercise.	①Athletes, especially those facing high training loads.<br>②Individuals with digestive issues or who need immune support.	Generally well tolerated; due to adaptive changes in response to the introduction of different bacterial species and subsequent fermentation activities in the gastrointestinal tract, gastrointestinal reactions may occur during the initial phase of supplementation.	①The effectiveness is strain-specific, so it is important to choose products backed by research for specific strains and dosages.<br>②Quality control and viable colony counts are key.
**Curcumin(20-24)**	①Exhibits anti-inflammatory propertiesthat may help alleviate post-exercise muscle soreness..<br>②Can lower exercise-induced oxidative stress to improve recovery.	①Possesses antioxidant and anti-inflammatory effects.<br>②May serve as an adjunct in managing chronic inflammatory conditions such as arthritis.	①Individuals require faster recovery after intense training sessions.<br>②Those with mild arthritis or chronic inflammation issues.	May result in gastrointestinal discomfort or other mild adverse reactions.	Curcumin has poor bioavailability on its own; it is often combined with black pepper (piperine) or other enhancers to improve absorption.

**Cherry juice(25-27)**	①May reduce recovery time after intense exercise.<br>②Can alleviate delayed onset muscle soreness and improve post-exercise condition.	①Antioxidant and anti-inflammatory properties may also contribute to improved sleep quality.<br>②Some studies suggest benefits for cardiovascular health.	①Endurance athletes or individuals engaged in prolonged training sessions.<br>②People focus on recovery and joint health.	①Its high sugar content might complicate blood sugar control.<br>②Some individuals might experience mild gastrointestinal discomfort.	①Total caloric intake should be considered.<br>②Monitor overall carbohydrate intake when using this supplement.

Reference

1. Andreux PA, Blanco-Bose W, Ryu D, Burdet F, Ibberson M, Aebischer P, et al. The Mitophagy Activator Urolithin a Is Safe and Induces a Molecular Signature of Improved Mitochondrial and Cellular Health in Humans. *Nat Metab* (2019) 1(6):595-603. Epub 20190614. doi: 10.1038/s42255-019-0073-4.

2. Liu S, D'Amico D, Shankland E, Bhayana S, Garcia JM, Aebischer P, et al. Effect of Urolithin a Supplementation on Muscle Endurance and Mitochondrial Health in Older Adults: A Randomized Clinical Trial. *JAMA Netw Open* (2022) 5(1):e2144279. Epub 20220104. doi: 10.1001/jamanetworkopen.2021.44279.

3. Singh A, D'Amico D, Andreux PA, Fouassier AM, Blanco-Bose W, Evans M, et al. Urolithin a Improves Muscle Strength, Exercise Performance, and Biomarkers of Mitochondrial Health in a Randomized Trial in Middle-Aged Adults. *Cell Rep Med* (2022) 3(5):100633. doi: 10.1016/j.xcrm.2022.100633.

4. Nishimoto Y, Fujisawa K, Ukawa Y, Kudoh M, Funahashi K, Kishimoto Y, et al. Effect of Urolithin a on the Improvement of Vascular Endothelial Function Depends on the Gut Microbiota. *Front Nutr* (2022) 9:1077534. Epub 20230105. doi: 10.3389/fnut.2022.1077534.

5. Jamialahmadi T, Hasanpour M, Vakilian F, Penson PE, Iranshahy M, Sahebkar A. Evaluation of Urolithin a Efficacy in Heart Failure Patients with Reduced Ejection Fraction: A Randomized, Double-Blind, Crossover, Placebo-Controlled Clinical Trial. *Rev Recent Clin Trials* (2024). Epub 20240220. doi: 10.2174/0115748871279354240209101604.

6. Denk D, Singh A, Kasler H, Boquet LA, D'Amico D, Gorol J, et al. Impact of Urolithin a Supplementation, a Mitophagy Activator on Mitochondrial Health of Immune Cells (Mitoimmune): A Randomized, Double-Blind, Placebo-Controlled Trial in Healthy Adults. *Journal of Clinical Oncology* (2024) 42(16_suppl):e14562-e. doi: 10.1200/JCO.2024.42.16_suppl.e14562.

7. Zhao H, Zhu H, Yun H, Liu J, Song G, Teng J, et al. Assessment of Urolithin a Effects on Muscle Endurance, Strength, Inflammation, Oxidative Stress, and Protein Metabolism in Male Athletes with Resistance Training: An 8-Week Randomized, Double-Blind, Placebo-Controlled Study. *J Int Soc Sports Nutr* (2024) 21(1):2419388. Epub 20241102. doi: 10.1080/15502783.2024.2419388.

8. Kreider RB, S. KD, Jose A, N. ZT, Robert W, Rick C, et al. International Society of Sports Nutrition Position Stand: Safety and Efficacy of Creatine Supplementation in Exercise, Sport, and Medicine. *Journal of the International Society of Sports Nutrition* (2017) 14(1):18. doi: 10.1186/s12970-017-0173-z.

9. Buford TW, Kreider RB, Stout JR, Greenwood M, Campbell B, Spano M, et al. International Society of Sports Nutrition Position Stand: Creatine Supplementation and Exercise. *Journal of the International Society of Sports Nutrition* (2007) 4(1):6.

10. Ostojic SM, Ahmetovic Z. Gastrointestinal Distress after Creatine Supplementation in Athletes: Are Side Effects Dose Dependent? *Res Sports Med* (2008) 16(1):15-22. doi: 10.1080/15438620701693280.

11. Antonio J, Candow DG, Forbes SC, Gualano B, Jagim AR, Kreider RB, et al. Common Questions and Misconceptions About Creatine Supplementation: What Does the Scientific Evidence Really Show? *Journal of the International Society of Sports Nutrition* (2021) 18:1-17.

12. Sanchez-Martinez J, Santos-Lozano A, Garcia-Hermoso A, Sadarangani KP, Cristi-Montero C. Effects of Beta-Hydroxy-Beta-Methylbutyrate Supplementation on Strength and Body Composition in Trained and Competitive Athletes: A Meta-Analysis of Randomized Controlled Trials. *J Sci Med Sport* (2018) 21(7):727-35. Epub 20171110. doi: 10.1016/j.jsams.2017.11.003.

13. Nissen SL, Sharp RL. Effect of Dietary Supplements on Lean Mass and Strength Gains with Resistance Exercise: A Meta-Analysis. *Journal of Applied Physiology* (2003).

14. Berton L, Bano G, Carraro S, Veronese N, Pizzato S, Bolzetta F, et al. Effect of Oral Beta-Hydroxy-Beta-Methylbutyrate (Hmb) Supplementation on Physical Performance in Healthy Old Women over 65 Years: An Open Label Randomized Controlled Trial. *PLoS One* (2015) 10(11):e0141757. Epub 20151103. doi: 10.1371/journal.pone.0141757.

15. Rawson ES, Miles MP, Larson-Meyer DE. Dietary Supplements for Health, Adaptation, and Recovery in Athletes. *International Journal of Sport Nutrition and Exercise Metabolism* (2018) 28(2):188-99. doi: 10.1123/ijsnem.2017-0340.

16. Holick MF, Binkley NC, Bischoff-Ferrari HA, Gordon CM, Hanley DA, Heaney RP, et al. Evaluation, Treatment, and Prevention of Vitamin D Deficiency: An Endocrine Society Clinical Practice Guideline. *The Journal of clinical endocrinology & metabolism* (2011) 96(7):1911-30.

17. Heimer M, Teschler M, Schmitz B, Mooren FC. Health Benefits of Probiotics in Sport and Exercise-Non-Existent or a Matter of Heterogeneity? A Systematic Review. *Frontiers in Nutrition* (2022) 9:804046.

18. de Paiva AKF, de Oliveira EP, Mancini L, Paoli A, Mota JF. Effects of Probiotic Supplementation on Performance of Resistance and Aerobic Exercises: A Systematic Review. *Nutrition Reviews* (2022) 81(2):153-67. doi: 10.1093/nutrit/nuac046.

19. Sotoudegan F, Daniali M, Hassani S, Nikfar S, Abdollahi M. Reappraisal of Probiotics' Safety in Human. *Food and Chemical Toxicology* (2019) 129:22-9.

20. McFarlin BK, Venable AS, Henning AL, Sampson JNB, Pennel K, Vingren JL, et al. Reduced Inflammatory and Muscle Damage Biomarkers Following Oral Supplementation with Bioavailable Curcumin. *BBA clinical* (2016) 5:72-8.

21. Salehi B, Stojanović-Radić Z, Matejić J, Sharifi-Rad M, Kumar NVA, Martins N, et al. The Therapeutic Potential of Curcumin: A Review of Clinical Trials. *European journal of medicinal chemistry* (2019) 163:527-45.

22. Campbell MS, A. CN, and Fleenor BS. Influence of Curcumin on Performance and Post-Exercise Recovery. *Critical Reviews in Food Science and Nutrition* (2021) 61(7):1152-62. doi: 10.1080/10408398.2020.1754754.

23. Hewlings SJ, Kalman DS. Curcumin: A Review of Its Effects on Human Health. *Foods* (2017) 6(10):92.

24. Heidari H, Bagherniya M, Majeed M, Sathyapalan T, Jamialahmadi T, Sahebkar A. Curcumin ‐ Piperine Co ‐ Supplementation and Human Health: A Comprehensive Review of Preclinical and Clinical Studies. *Phytotherapy Research* (2023) 37(4):1462-87.

25. Santos HO, Genario R, Gomes GK, Schoenfeld BJ. Cherry Intake as a Dietary Strategy in Sport and Diseases: A Review of Clinical Applicability and Mechanisms of Action. *Critical Reviews in Food Science and Nutrition* (2021) 61(3):417-30.

26. McHugh MP. "Precovery" Versus Recovery: Understanding the Role of Cherry Juice in Exercise Recovery. *Scandinavian journal of medicine & science in sports* (2022) 32(6):940-50.

27. Elliot DL, Kuehl KS, Jones KD, Dulacki K. Using an Eccentric Exercise-Testing Protocol to Assess the Beneficial Effects of Tart Cherry Juice in Fibromyalgia Patients. *Integr Med* (2010) 9:24-9.
